# Reduced‐dose chemotherapy followed by blinatumomab in induction therapy for newly diagnosed B‐cell acute lymphoblastic leukemia

**DOI:** 10.1002/cam4.7062

**Published:** 2024-03-16

**Authors:** Jing Lu, Huifen Zhou, Xin Zhou, Yonggong Yang, Laigen Tong, Miao Miao, Xiaofei Yang, Suning Chen

**Affiliations:** ^1^ Department of Hematology The First Affiliated Hospital of Soochow University, Jiangsu Institute of Hematology, National Clinical Research Center for Hematologic Diseases Suzhou China; ^2^ Department of Hematology Wuxi People's Hospital Affiliated to Nanjing Medical University WuXi China; ^3^ Department of Hematology The Affiliated Drum Tower Hospital of Nanjing University Medical School Nanjing China; ^4^ Yixing People's Hospital, The Affiliated Hospital of Jiangsu University Yixing China

**Keywords:** B‐cell acute lymphoblastic leukemia, blinatumomab, induction therapy, reduced‐dose chemotherapy

## Abstract

**Background:**

Blinatumomab early‐line treatment in B‐cell precursor acute lymphoblastic leukemia (B‐ALL) might improve clinical outcomes.

**Methods:**

We conducted a retrospective real‐world cohort analysis in 20 newly diagnosed B‐ALL patients who received reduced‐dose chemotherapy (idarubicin, vindesine, and dexamethasone) for 1–3 weeks, followed by blinatumomab for 1–4 weeks as an induction therapy.

**Results:**

At the end of the induction therapy, a complete remission rate of 100% was achieved; 17 (85%) patients were minimal residual disease (MRD) negative (<1 × 10^−4^). Adverse events (AEs) were reported in 12 (60%) patients—43.8% were grade 1–2 and 56.2% were grade 3–4. No incidence of neurotoxicity or grade ≥3 cytokine release syndrome was reported.

**Conclusions:**

Blinatumomab demonstrated a significant improvement in clinical outcomes in patients with newly diagnosed B‐ALL irrespective of their poor‐risk factor status and the pretreatment blast burden.

## 1 INTRODUCTION

For nearly 30 years, induction regimens for adult patients with B‐cell acute lymphoblastic leukemia (B‐ALL) have mainly been modified based on pediatric multidrug combination chemotherapy regimens.^1^ The hyper‐fractionated cyclophosphamide, vincristine, adriamycin, and dexamethasone (Hyper‐CVAD) and pediatric Augmented Berlin‐Frankfurt‐Münster (ABFM) are two established treatment regimens with convincing favorable outcomes in B‐ALL,^2^ though Hyper‐CVAD is associated with a better safety profile compared to ABFM.^3^ However, the depth of remission with currently available regimens is so limited that half of the adult patients with B‐ALL relapse leading to poor survival outcomes.^4^, ^5^ Therefore, balancing the efficacy and safety of treatment for adult B‐ALL patients remains a major challenge. The advent of targeted and immunotherapies has changed the landscape of B‐ALL treatment. The use of tyrosine kinase inhibitor (TKI) in Philadelphia chromosome–positive B‐ALL (Ph + B‐ALL) induction therapy has substantially reduced the intensity of chemotherapy and related mortality while increasing the remission rate and thereby improving the prognosis.^6^ With the addition of immunotherapeutic agents such as blinatumomab, a bispecific anti‐CD3 and anti‐CD19 monoclonal antibody, patients with relapsed or refractory as well as minimal residual disease (MRD)‐positive B‐ALL have also achieved a higher molecular response and longer survival.^7^, ^8^ The approval of blinatumomab is an important milestone especially for the treatment of relapsed or refractory Philadelphia chromosome–negative B‐ALL (Ph − B‐ALL), a subtype that lacks the molecular targets for targeted therapies.^9^, ^10^ Encouraging clinical efficacy data have also been observed for blinatumomab in the early‐line setting as sequential therapy in B‐ALL.^11^ In current study, we evaluated the efficacy and safety profile of blinatumomab in induction therapy of newly diagnosed patients with B‐ALL.

## 2 METHODS

This retrospective study consecutively included newly diagnosed patients with B‐ALL who had received blinatumomab as the initial induction therapy at four hematology treatment centers in Jiangsu Province in China between December 2021 and October 2022. Before blinatumomab, all patients were treated with a small amount of chemotherapy or corticosteroid, and we defined this pretreatment as reduced‐dose chemotherapy (RDC). The RDC treatment period spanned for 1–3 weeks and contained idarubicin (IDA), vindesine (VDS), and dexamethasone (DEX), which were administered at a dose of 8 mg/m^2^ for IDA (median dose: 0; range, 0–3), 3 mg/m^2^ for VDS (median dose: 1; range, 0–3), and 9 mg/m^2^/day for DEX (median duration: 7 days; range, 0–21). Blinatumomab was given at a dose of 9 μg/day for the first 7 days and 28 μg/day for the remaining days (median duration: 28 days; range, 7–28).This study was approved by institute's ethics committee and was conducted in accordance with Declaration of Helsinki.

Efficacy outcomes included complete remission (CR) and MRD negativity. Safety data including cytokine release syndrome (CRS) and neurological symptoms were collected. Most bone marrow evaluations were performed after 2 weeks of blinatumomab treatment, and response criteria for blood and bone marrow were defined according to the National Comprehensive Cancer Network Guidelines (Version 1.2022). MRD was assessed by multicolor flow cytometry, and the threshold of MRD was 1 × 10^−4^. Neutrophil and platelet deficiencies were defined as count <0.5 × 10^9^/L and < 20 × 10^9^/L, respectively. The grading of adverse events (AEs) was based on the Common Terminology Criteria for Adverse Events Version 5.0. CRS and neurotoxicity were evaluated according to the American Society for Transplantation and Cellular Therapy Consensus Grading.^12^

## 3 RESULTS

**FIGURE 1:**
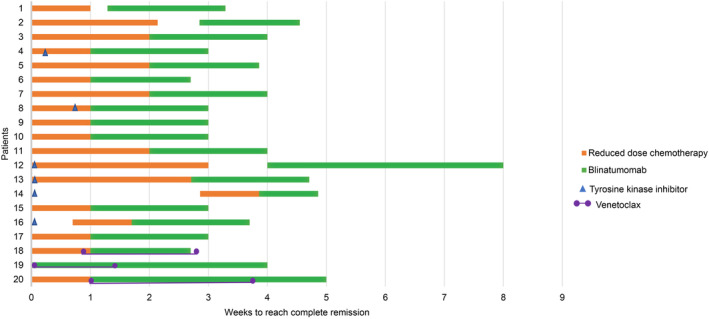
Process and response of induction therapy.

Overall, 20 patients were enrolled; Ph − B‐ALL was the most common subtype diagnosed in 11 (55%) patients. The median age of the cohort was 44 years (range, 8–92; Supplementary Table S1). Twelve (60%) patients were identified with a risk factor; *BCR::ABL1* was the most common risk factor found in six (50%) patients (Table 1). All patients received RDC followed by blinatumomab as induction regimen. The reasons for patients not completing the conventional first‐line chemotherapy included chemotherapy‐related toxicities (4/20; sepsis [*n* = 1], infectious shock [*n* = 1] and pneumonia [*n* = 2]), chemotherapy insensitivity (3/20), severe comorbidity or underlying disease at initial diagnosis (10/20), and patient‐requested chemo‐free therapy (3/20; Table 1). For patients with Ph + B‐ALL (*n* = 6), TKI was continuously used after the detection of Philadelphia chromosome. Three patients with mixed‐phenotype acute leukemia (MPAL) had 10–19 days of venetoclax added to their induction therapy (Table 1). Some patients had an interval before or after the RDC treatment due to an infection or waiting for the results of bone marrow evaluation (Figure 1).

**TABLE 1:** Baseline characteristics and induction therapy data at patient level.

Gender/age (years)	Diagnosis^a^	Poor‐risk factors^b^	Reasons for Blina to replace Chemo	Blasts burden (%)	Induction treatment					Outcomes for induction treatment				Complications (grade)				
					RDC^c^					Disease status		Days of Blina		AEs	CRS	Days of myelosuppression		
					IDA (dose)	VDS (dose)	DEX (days)	Blina^d^ (days)	Other drugs	Prior Blina	Post Blina	Reach CR	Reach MRD‐	During RDC	During Blina		ANC <0.5 × 10^9^/L	Platelets <20 × 10^9^/L
F/63	B‐ALL	IKZF1 del	Chemo‐associated sepsis	83	1	1	7	14	No	N/A	CR, MRD‐	14	14	Sepsis (3)	Fever (1)	1	14	11
M/43	B‐ALL	No	Chemo‐associated infectious shock	83	3	3	15	17	No	PR	CR, MRD‐	12	12	Infectious shock (4)	No	No	41	48
M/44	B‐ALL	Hypodiploidy, TP53	Chemo‐associated pneumonia	61.5	2	2	14	19	No	N/A	CR, MRD‐	14	19	Pneumonia (3)	Fever (1)	No	18	25
M/28	Ph + B‐ALL	BCR::ABL1	Patient‐requested Chemo‐free	95.5	0	0	7	28	Imatinib	N/A	CR, MRD‐	14	14	No	Fever (1)	No	3	9
M/35	B‐ALL	No	Chemo‐associated pneumonia	53.6	2	2	14	26	No	MLFS, MRD+	CR, MRD‐	13	13	Pneumonia (3)	No	No	0	0
M/45	B‐ALL	No	Initially with severe perianal infection	78.9	0	1	7	28	No	N/A	CR, MRD‐	12	12	No	Fever (3)	No	1	0
M/32	B‐ALL	KMT2A rearranged	Patient‐requested Chemo‐free	78.5	0	1	14	21	No	N/A	CR, MRD‐	14	14	No	Pneumonia (3)	1	1	0
F/60	Ph + B‐ALL	BCR::ABL1	Underlying disease	92.5	0	0	7	14	Imatinib	MLFS, MRD+	CR, MRD‐	14	14	No	No	No	5	7
F/13	B‐ALL	No	Chemo‐insensitivity	94	0	1	7	28	No	NR	CR, MRD‐	14	14	No	No	1	10	0
M/14	B‐ALL	No	Initially with infectious shock	82.7	0	1	7	28	No	PR	CR, MRD‐	14	14	No	No	1	10	2
M/8	B‐ALL	No	Chemo‐insensitivity	98	2	2	14	14	No	NR	CR, MRD‐	14	14	No	No	1	13	4
M/29	Ph + B‐ALL	BCR::ABL1	Chemo‐insensitivity	80	0	3	21	28	Olverembatinib	PR	CR, MRD‐	28	28	No	No	No	25	0
F/66	Ph + B‐ALL	BCR::ABL1	Initially with coagulation disorder	95.2	0	0	19	28	Olverembatinib	PR	CR, MRD‐	14	14	No	No	1	18	16
M/92	Ph + B‐ALL	BCR::ABL1	Underlying disease and old age	93.5	0	0	7	7	Olverembatinib	N/A	CR, MRD+	7	Not reached	No	Fatigue (2)	No	6	0
F/38	B‐ALL	No	Patient‐requested Chemo‐free	85.5	0	1	7	28	No	N/A	CR, MRD‐	14	14	No	Fever (1)	No	0	0
M/64	Ph + B‐ALL	BCR::ABL1	Underlying disease	20.5	0	0	7	28	Olverembatinib	N/A	CR, MRD‐	14	14	No	Atrial fibrillation (3) ALT (2)	No	0	0
F/53	B‐ALL	No	Underlying disease	82.5	1	1	7	14	No	N/A	CR, MRD‐	14	14	No	No	2	4	0
M/50	MPAL, B/myeloid	KMT2A rearranged	Initially with pneumonia and DIC	59.9	0	0	7	28	VEN	N/A	CR, MRD+	12	Not reached	No	No	No	23	14
F/60	MPAL, B/myeloid	FLT3/ITD, RUNX1	Initially with pneumonia	87	0	0	0	28	VEN	N/A	CR, MRD‐	28	28	No	ALT (2)	No	43	51
M/43	MPAL, B/myeloid	KMT2A rearranged	Initially with severe perianal infection	52	0	0	7	28	VEN	NR	CR, MRD+	28	Not reached	No	Pneumonia (3) GIB (3)	No	39	21

Abbreviations: AE, adverse event; ALL, acute lymphoblastic leukemia; ALT, alanine aminotransferase; ANC, absolute neutrophil count; Blina, blinatumomab; B‐ALL, B‐cell ALL; Ph + B‐ALL, Philadelphia chromosome–positive B‐ALL; Chemo, chemotherapy; CR, complete remission; CRS, cytokine release syndrome; del, deletion; DEX, dexamethasone; DIC, disseminated intravascular coagulation; GIB, gastrointestinal bleeding; IDA, idarubicin; MLFS, morphologic leukemia free state; MPAL, mixed‐phenotype acute leukemia; MRD, minimal residual disease; NCCN, National Comprehensive Cancer Network; NR, no remission; N/A, not available; PR, partial remission; RDC, reduced‐dose chemotherapy; TKI, tyrosine kinase inhibitor; VDS, vindesine; VEN, venetoclax.

^a^ All the patients were diagnosed according to the 2016 WHO classification of Tumours of Haematopoietic and Lymphoid Tissues.

^b^ The poor‐risk factors of ALL were identified according to the NCCN guideline for ALL (Version 1, 2022), while the poor‐risk factors of MPAL also refer to the NCCN guideline for AML (Version 1, 2022).

^c^ The dosage of RDC during induction treatments is 8 mg/m^2^/dose for IDA, 3 mg/m^2^/dose for VDS, and 9 mg/m^2^/day for DEX.

^d^ The dosage of Blina during induction treatments is 9 μg/day for the first 7 days and 28 μg/day for the rest days.

The median white blood cell count, hemoglobin content, and platelet count at an initial diagnosis were 6.2 × 10^9^/L (range, 0.9–362.5), 89 g/L (range, 38–165), and 67 × 10^9^/L (range, 6–279), respectively. The median percentage of blasts in bone marrow at the initial diagnosis was 82.9% (range, 20.5–98; Table 1 and Supplementary Table S1).

Nine (45%) patients underwent bone marrow evaluation before treatment with blinatumomab, and none of the RDC treatments resulted in CR. Nevertheless, at the end of the induction therapy, all 20 (100%) patients achieved CR after a median duration of 25 days (range, 19–56), and 17 (85%) patients achieved MRD negativity (Supplementary Table S1). A 92‐year‐old patient (who discontinued blinatumomab after 7 days due to fatigue) and 2 patients with MPAL (their lowest MRD was 2.6 × 10^−4^ and 2.5 × 10^−3^, respectively) did not achieve MRD negativity (Table 1). Further, of the 6 Ph + B‐ALL patients with *BCR::ABL1*, 2 achieved complete molecular response (*BCR::ABL1* negative by quantitative PCR) at the end of initial induction treatment and other 4 achieved it on subsequent treatments.

The median duration of blinatumomab infusion before achieving CR or MRD negativity was 14 days for both outcomes (range, 7–28 for CR and 12–28 for MRD negativity; Table 1). After the induction therapy, patients were treated with either multidrug combination chemotherapy (based on high‐dose cytarabine or methotrexate) or blinatumomab (28 days per cycle) as consolidation regimens. The median cycles for consolidation therapy were 2 cycles (range, 1–7). By November 2022, only one patient had a bone marrow relapse at Day 254, and the median follow‐up was 103 days (range, 31–334) with 100% overall survival.

In total, 12 of 20 (60%) patients had at least 1 AE during the induction therapy. A total of 16 AEs were reported, including 43.8% of grade 1–2 and 56.3% of grade 3–4 (Supplementary Table S1). All AEs during RDC were of grade 3–4, and grade 3–4 AEs were observed in 5 of 12 (41.6%) patients during blinatumomab treatment (Table 1). No patients had any unexpected serious AEs. Blinatumomab‐related CRS was reported in seven (35%) patients, and all of them were grade 1–2 (Table 1). No incidence of neurotoxicity was reported. The median duration of neutrophil deficiency during the induction treatment was 10 days (range, 0–43), and the platelet deficiency was 3 days (range, 0–51; Table 1). The median time taken by patients to recover from RDC‐related hemocytopenia was 5 days (range, −1–35) for neutrophil recovery and 6 days (range, 2–35) for platelet transfusion independence.

## 4 DISCUSSION

Given the success of blinatumomab in treating relapsed or refractory B‐ALL, it has been proposed that the addition of blinatumomab to chemotherapy in the early‐line setting may further improve long‐term prognosis.^11^ However, the clinical outcomes of treatment in real‐world may differ outside of the controlled settings of clinical trials.^13^ Therefore, this real‐world study was conducted to evaluate the efficacy and toxicity of blinatumomab in newly diagnosed patients with B‐ALL and summarize the experience of using blinatumomab.

Overall, the efficacy of blinatumomab in the real‐world setting was consistent with that reported in clinical trials. The results from our study were in line with a phase 2 trial that evaluated the use of hyper‐CVAD in sequential combination with blinatumomab in newly diagnosed patients with B‐ALL and reported a 100% CR and MRD negativity of 96%.^11^ Also, the induction regimen in our study demonstrated a comparable efficacy to mini‐hyper‐CVD + inotuzumab ozogamicin +/− blinatumomab regimen that resulted in overall response rate of 91% and MRD‐negativity rate of 84% after 1 cycle of induction therapy in a phase 2 trial in newly diagnosed patients with B‐ALL, but blinatumomab appeared to be better tolerated (inotuzumab ozogamicin related grade 3–4 liver toxicities were observed in 26.2% patients and veno‐occlusive disease was observed in 8% patients).^14^ The use of RDC followed by blinatumomab as the induction therapy in our study was a better drug combination for newly diagnosed patients with B‐ALL. With the early use of blinatumomab, patients with Ph + B‐ALL could obtain CR and MRD negativity more rapidly, which showed an advantage compared with the phase 2 D‐ALBA trial from the Italian GIMEMA Group (median duration of induction: 25 vs. 85 days).^9^ The clinical trial SWOG 1318, where blinatumomab was used as a single‐agent induction therapy in newly diagnosed patients with Ph−B‐ALL,^15^ reported an overall response rate of only 66% that was far less effective than that of multidrug combination chemotherapy. Furthermore, in another clinical trial of chemotherapy combined with blinatumomab, ALL08, the chemotherapy dose was not substantially reduced, and hence, the incidence of chemotherapy‐related AEs remained high.^16^ The trial ALL08 reported 53 episodes of sepsis, infection or febrile neutropenia and 1 grade 3 CRS versus 2 episodes of sepsis and infectious shock and no grade 3 CRS in our study. Moreover, no incidence of neurotoxicity was reported in our study compared with seven episodes in the ALL08 study^16^ and one episode of neurological disorder in the D‐ALBA study.^9^ The incidence of overall grade 3 events was also less in our study (8 events) compared with the D‐ALBA study (21 events). Hence, our induction therapy study found a good balance between chemotherapy and immunotherapy, minimizing the chemotherapy‐related complications and increasing the efficacy of induction therapy. After a median duration of 1 week of RDC followed by 14 days of blinatumomab, 100% CR rate was achieved along with an early MRD negativity in 85% of patients, which was very important to reduce the recurrence rate and prolong survival.^17^, ^18^ Notably, there were only three cases of MPAL, and our study was the first to use venetoclax in combination with blinatumomab as the induction therapy in such patients, which achieved a very good efficacy (100% CR). Moreover, despite 60% of patients presenting with one or the other poor‐risk factors and a high blast burden of 82.9% at baseline in our study, 100% CR was achieved with blinatumomab. Therefore, blinatumomab was well tolerated and effective in the treatment of newly diagnosed patients with B‐ALL, irrespective of their poor‐risk factor status and high blast burden.

Our study was limited by its small sample size, single‐arm design, and relatively short follow‐up. Owing to these limitations, we are conducting a study (NCT05557110) in newly diagnosed nonelderly patients with Ph−B‐ALL to further validate the efficacy of RDC followed by blinatumomab as the induction therapy.

To conclude, this is a retrospective real‐world study that evaluated the efficacy and safety of blinatumomab in newly diagnosed B‐ALL. This study supports the use of blinatumomab for the early treatment of B‐ALL and summarized the dose balance between chemotherapy and blinatumomab, which could help improve survival outcomes and limit chemotherapy‐related toxicity.

## AUTHOR CONTRIBUTIONS

**Jing Lu:** Formal analysis (equal); methodology (lead); writing – original draft (lead). **Huifen Zhou:** Data curation (equal); formal analysis (equal); investigation (equal). **Xin Zhou:** Data curation (equal); formal analysis (equal); investigation (equal). **Yonggong Yang:** Data curation (equal); formal analysis (equal); investigation (equal). **Laigen Tong:** Data curation (equal); formal analysis (equal); investigation (equal). **Miao Miao:** Conceptualization (equal); formal analysis (equal); project administration (equal); writing – review and editing (equal). **Xiaofei Yang:** Conceptualization (equal); formal analysis (equal); project administration (equal); writing – review and editing (equal). **Suning Chen:** Conceptualization (equal); formal analysis (equal); project administration (equal); writing – review and editing (equal).

## FUNDING INFORMATION

This research received no specific grant from any funding agency in the public, commercial, or not‐for‐profit sectors.

## CONFLICT OF INTEREST STATEMENT

The authors have no relevant financial or non‐financial interests to disclose.

## ETHICAL APPROVAL STATEMENT

This study was performed in line with the principles of the Declaration of Helsinki. Approval was granted by the Committee on Medical Ethics of the First affiliated Hospital of Soochow University (Approval number: 2023010).

## PATIENT CONSENT STATEMENT

Written informed consent was obtained from all participants involved in this study. All participants received a detailed explanation of the study's objectives and procedures, and they voluntarily provided written consent for their participation. The study was conducted in accordance with the applicable ethical guidelines and regulations, and all personal information was handled with strict confidentiality.

## PERMISSION TO REPRODUCE MATERIAL FROM OTHER SOURCES

Not applicable.

## Supporting information


Table S1.

## Data Availability

All data generated or analyzed during this study are included in this published article (and its Supporting Information files).
