# Supplementary material for: Reduced‐dose chemotherapy followed by blinatumomab in induction therapy for newly diagnosed B‐cell acute lymphoblastic leukemia
Source: Cancer Med. 2024 Mar 16;13(5):e7062. doi: 10.1002/cam4.7062 (PMC10943272; doi:10.1002/cam4.7062)
Supplement: Supplementary file 1 — Table S1. [file CAM4-13-e7062-s001.docx]

**SUPPLEMENTARY TABLE 1.** Baseline characteristics and induction therapy outcomes at cohort level

| Baseline characteristics | |  |
| --- | --- | --- |
| Characteristic | **Number of patients (N=20)** | |
| Age years, median (range) | 44.0 (8-92) | |
| Gender, n (%) |  |  |
| Male | 13 (65) | |
| Female | 7 (35) | |
| B-ALL subtypes^*^, n (%) |  |  |
| Ph- B-ALL | 11 (55) | |
| Ph+ B-ALL | 6 (30) | |
| MPAL, B/myeloid | 3 (15) | |
| Poor risk factors^#^ |  |  |
| No | 8 (40) | |
| Yes | 12 (60) | |
| BCR::ABL1 | 6 (50) | |
| Others | 6 (50) | |
| Blast burden %, median (range) | 82.9 (20.5-98) | |
| <50%, n (%) | 1 (5) | |
| 50% to <75% | 4 (20) | |
| ≥75%, n (%) | 15 (75) | |
| Concomitant treatment along with induction therapy, n (%) |  |  |
| No | 11 (55) | |
| Yes | 9 (45) | |
| TKI | 6 (67) | |
| Venetoclax | 3 (33) | |
| Efficacy outcomes (N=20) | | |
| Prior to blinatumomab treatment, n (%) |  |  |
| CR | 0 | |
| PR | 4 (20) | |
| MLFS, MRD+ | 2 (10) | |
| NR/NA | 14 (70) | |
| After blinatumomab treatment, n (%) |  |  |
| CR | 20 (100) | |
| MRD- | 17 (85) | |
| Adverse events (N=20) | | |
| AEs, n (%) | **Any grade** | **≥ Grade 3** |
| Fever | 5 (25) | 1 (5) |
| Pneumonia | 4 (20) | 4 (20) |
| Increased ALT | 2 (10) | 0 |
| Sepsis | 1 (5) | 1 (5) |
| Infectious shock | 1 (5) | 1 (5) |
| Atrial fibrillation | 1 (5) | 1 (5) |
| Gastrointestinal bleeding | 1 (5) | 1 (5) |
| Fatigue | 1 (5) | 0 |

*All the patients were diagnosed according to the 2016 WHO classification of Tumours of Haematopoietic and Lymphoid Tissues. ^#^ The poor risk factors of ALL were identified according to the NCCN guideline for ALL (Version 1, 2022), while the poor risk factors of MPAL also refer to the NCCN guideline for AML (Version 1, 2022). AE, adverse events; ALT, alanine aminotransferase; ALL, acute lymphoblastic leukemia; B-ALL, B-cell ALL; CR, complete remission; MLFS, morphologic leukemia-free state; MPAL, mixed-phenotype acute leukemia**;** MRD, minimal residual disease; N/A, not available; NR, no remission**;** Ph , Philadelphia chromosome; PR, partial remission
